# Prenatal exposure of diclofenac sodium alters the behavioral development of young Wistar rats

**DOI:** 10.3906/biy-1904-33

**Published:** 2019-10-14

**Authors:** Birsen ELİBOL, Begüm ARITAN OĞUR, Havva DOĞRU

**Affiliations:** 1 Department of Medical Biology, Faculty of Medicine, Bezmiâlem Vakıf University, İstanbul Turkey; 2 Department of Psychiatry, Faculty of Medicine, Gülhane Medical Hospital, Ankara Turkey; 3 Department of Biological Sciences, Faculty of Arts and Science, Middle East Technical University, Ankara Turkey

**Keywords:** Diclofenac sodium, anxiety, learning and memory, sensory motor tests, rat

## Abstract

Diclofenac sodium (DS), a potent inhibitor of cyclooxygenase, reduces the release of arachidonic acid and formation of prostaglandins. Being a nonsteroid drug that shows antiinflammatory action, the possible side effects of fetal DS administration gain importance in public and medical applications. Herein, the effects of DS administration (1 mg/kg) during gestational days 5–20 were investigated on the performance of Wistar rat pups in a variety of behavioral tasks. Four-week-old pups were subjected to sensory motor tests, a plus maze, an open field, the Morris water maze, and a radial arm maze. Fetal DS disrupted some sensory motor performances, such as visual placing and climbing in both females and males. In the open field, DS females had a higher level of anxiety and male DS pups habituated to the environment slowly compared to controls. The DS pups showed slower rates of learning, whereas no substantial between-group differences were found in the performance of spatial memory compared to both controls. Furthermore, working memory was negatively affected by fetal DS. In conclusion, it was indicated that DS administration during pregnancy had slight behavioral impacts with a delay in learning and a defect in the short-term memory in juvenile rats.

## 1. Introduction

Diclofenac sodium (DS; sodium-(O-[(2,6-dichlorophenyl)-amino]-phenyl)-acetate) acts as a potent cyclooxygenase (COX) inhibitor, reducing arachidonic acid release and formation of prostaglandins (Capone et al., 2007). It is a common nonsteroidal type drug that has ameliorative effects on inflammation, pain, and pyrexia. Clinically, DS is often used as a pain-killer, fever-reducer, and antiinflammatory agent for different types of arthritis, acute gout, menorrhagia, postoperative pain after surgery, etc. However, it has been reported that in adults, taking DS as a medication may produce some side effects such as serious bleeding in the upper gastrointestinal system, dysfunction in the cardiovascular system, failure in the functions of platelets, and convulsions (Russell, 2001; Liu et al., 2005; Andersohn et al., 2006; Capone et al., 2007). In the adult brain, DS was reported to inhibit neural stem cell differentiation and proliferation (Kudo et al., 2003).

In addition to its side effects in adult use, DS also has some teratogenic effects. For instance, there are reports about the adverse effects of DS administration on organogenesis taking place during the early period of gestation (Carp et al., 1988; Chan et al., 2001; Kudo et al., 2003; Siu and Lee, 2004). Although its known that DS crosses the human placenta in the second trimester of gestation (Ostensen, 1998; Siu et al., 2000), there is much less data concerning the potential side effects of DS administration at more advanced stages of pregnancy and almost no data about the developmental effects of DS on the central nervous system and future behavior. A limited study on the usage of antiinflammatory drugs and behavioral alterations showed that diclofenac sodium administration during adult life prevents repeated stress-induced memory deficits, which can occur due to oxidative stress-related inflammation (Emad et al., 2017). In addition, a previous study demonstrated that the use of DS can prevent a depression-like phenotype (De La Garza and Asnis, 2003) and alter reward behavior (De La Garza et al., 2004) in rats.

Taking this into consideration, the present study aimed to investigate the effects of gestational DS administration on the performance of Wistar rat pups in a variety of behavioral tasks.

## 2. Materials and methods

### 2.1. Subjects and DS administration

Adult (150–200 g) female (n = 20) and male (n = 8) Wistar rats (Hıfzıssıhha Serum-Production Facility, Ankara, Turkey) were used in the present study. The experimental protocol of this study was approved by the local scientific ethics committee. Standard animal housing conditions (22 °C temperature and 60% humidity) were applied to rats with ad libitum food and water during 12-h daily light/dark periods. During the mating period, observation of a vaginal plug in a rat was considered as evidence of successful fertilization and that day was assigned as gestational day 0 (GD0). On GD5, pregnant dams were separated to one of three treatment groups: diclofenac sodium group (D), pair-fed sham control group (C), and pure control group (PC).

DS (Voltaren, 75 mg/3 mL ampule) in a dose of 1 mg/kg/day, as adapted from previous studies (Ragbetli et al., 2007; Odaci et al., 2010; Zengin et al., 2013), was subcutaneously injected to the D group of pregnant dams beginning from GD5 to GD20. In addition, 1 mL of physiological serum was also subcutaneously injected to the pregnant C dams throughout the same time period. The pregnant rats in the PC group were only weighed daily. In the weaning period, male and female pups of all treatment groups were subjected to behavioral tests. 

### 2.2. Behavioral tests

At the end of the 4th week of age, all male and female pups were subjected to a series of behavioral tasks including sensorimotor tests, activity/anxiety open field test, anxiety plus maze test, and learning/memory tasks: a 12-arm radial maze (RAM) and the Morris water maze (MWM), both run under allo- and idiothetic stimulus conditions and traced and analyzed with a computerized video tracking system (EthoVision, Noldus Information Technology, the Netherlands) or by an observer.

#### 2.2.1. Sensory motor tests

There were 8 different parameters to determine the sensory motor functions of rat pups, as follows: 

1) Visual placing: The pup was lifted from its back and slowly moved toward the edge of a table without allowing its whiskers to touch the edge. A positive score was given if the pup reached and extended forepaws towards the table.

2) Walking initiation test: The pup was placed on a horizontal surface of a table. The time taken to move one body length on the table surface was recorded (Horvath et al., 2015). 

3) Turning on an inclined screen: The pup was placed on the center of a horizontal wire mesh. It was gently inclined to 45° with the pup facing downward and the pup’s latency to turn upward was measured.

4) Wire hanging: The pup was placed on a horizontal wire with its front paws. The time until the pup fell was recorded.

5) Clasping the limbs: The pup was suspended by its tail. If its hindlimbs and forelimbs extended away from its body, it was considered as a positive score.

6) Climbing a vertical screen: The pup was put at the center of a vertical wire mesh. The time until the pup reached the top was recorded.

7) Swimming skills: The pup was placed in a water tank and the swim velocity of the pup was recorded.

#### 2.2.2. Elevated plus maze

The apparatus has a central area (10 × 10 cm) and two closed (50 × 10 × 40 cm) and two open (50 × 10 cm) arms, elevated 50 cm above the floor. At the testing session, each animal was put in the central area. For 5 min, the total duration of time that the animal spent in the open and closed arms was measured to determine the anxiety level of each animal by video tracking system (Kayir and Uzbay, 2006). Before putting another rat in the maze, the apparatus was cleaned with 70% ethanol solution and a sufficient length of time was waited until no liquid was apparent on the arms of maze (approximately 30 s) to reduce odor cues from the previous rat.

#### 2.2.3. Open field test

The apparatus was formed with a square platform of 120 × 120 cm with walls that were 30 cm high. The pup was placed in the apparatus facing the wall. The locomotor activity of each pup, either in the peripheral zone or in the central zone of the field, was recorded by the EthoVision animal tracking system. In addition to the system’s records of distance moved (ambulation) and time spent in each of the zones, behavioral parameters like rearing, sniffing, grooming, freezing, and number of defecations were also determined during 10 min. The maze was cleaned with 70% ethanol before putting the next rat in the maze. 

#### 2.2.4. Morris water maze test (MWM)

A circular MWM tank (150 cm × 60 cm) was used to monitor the spatial learning and memory of the pups. It was filled with water containing nontoxic paint and the temperature of the water was maintained at 23 °C by an automatic heater. A computerized video tracking system was used to observe the rat pup in the pool while it was finding the hidden platform (11 cm × 11 cm) according to extra-maze cues. Two different strategies were applied to the rat pups for place learning. During each trial, in place learning (allothetic paradigm), environmental cues in the room as spatial reference points were used by the animals, or in the idiothetic paradigm, these cues were blocked by nontransparent screens surrounding the pool (Elibol-Can et al., 2014). During place learning, rats were given four daily trials, for 4 consecutive days (allothetic paradigm) or 6 consecutive days (idiothetic paradigm). The trial was stopped when the platform was found by the rat pup or when 60 s had passed. All the movements of pups (swim velocity, distance and trajectory, and escape latency) were recorded by the video tracking system.

In the probe trial, which measured the strength of the acquired place preference, the pups were swam for 60 s in the pool without a platform. The percentages of the distance swum and time spent in the platform quadrant and in an imaginary circle (40 cm in diameter) around the platform called annulus 40 were recorded.

#### 2.2.5. Radial arm maze test (RAM)

A flat gray plywood 12-arm radial maze (80 cm above the floor) was used to assess the memory of pups. The experimental room was furnished with different distal visual cues. Each arm of maze (60 cm × 9 cm) had sidewalls made by transparent Plexiglas 15 cm in height to block the pups from directly crossing from one arm to another and to allow them to see the visual cues in the room. The maze had a central platform (40 cm in diameter) and was separated from each arm by a guillotine door. At the end of each arm there was a food well (2 cm wide and 2 cm deep). During the RAM training, six out of twelve arms were semirandomly baited with equal amounts of chocolate rice puffs. The baited arms were the same all throughout the training. On each trial, pups were placed at the center of the maze and the guillotine doors leading to the arms were raised, and the rats tried to choose the correct arms. Each trial was completed when the rat: 1) found all pellets, 2) made 12 choices, or 3) spent the maximum duration (10 min). Reference memory error was noted when the rat entered an unbaited arm and working memory error was noted when the rat reentered a baited arm via the monitor of the computerized video tracking system by an observer. The criterion was to make 5 out of the first 6 choices be the baited arms in three consecutive daily trials. 

### 2.3. Statistical analysis

For all behavioral parameters, group means and standard errors of means (SEMs) were calculated. Repeated measures ANOVAs with group as independent factor and sessions as repeated measures were used for behavioral MWM data. For the multiple comparisons, a post hoc test (Fisher’s least significant difference (LSD)) was used. Additionally, one-way ANOVA with group as an independent factor was applied to the experimental data. The statistical analyses were performed using SPSS and P ≤ 0.05 was considered statistically significant.

## 3. Results

### 3.1. Sensory motor tests

**Figure 1:**
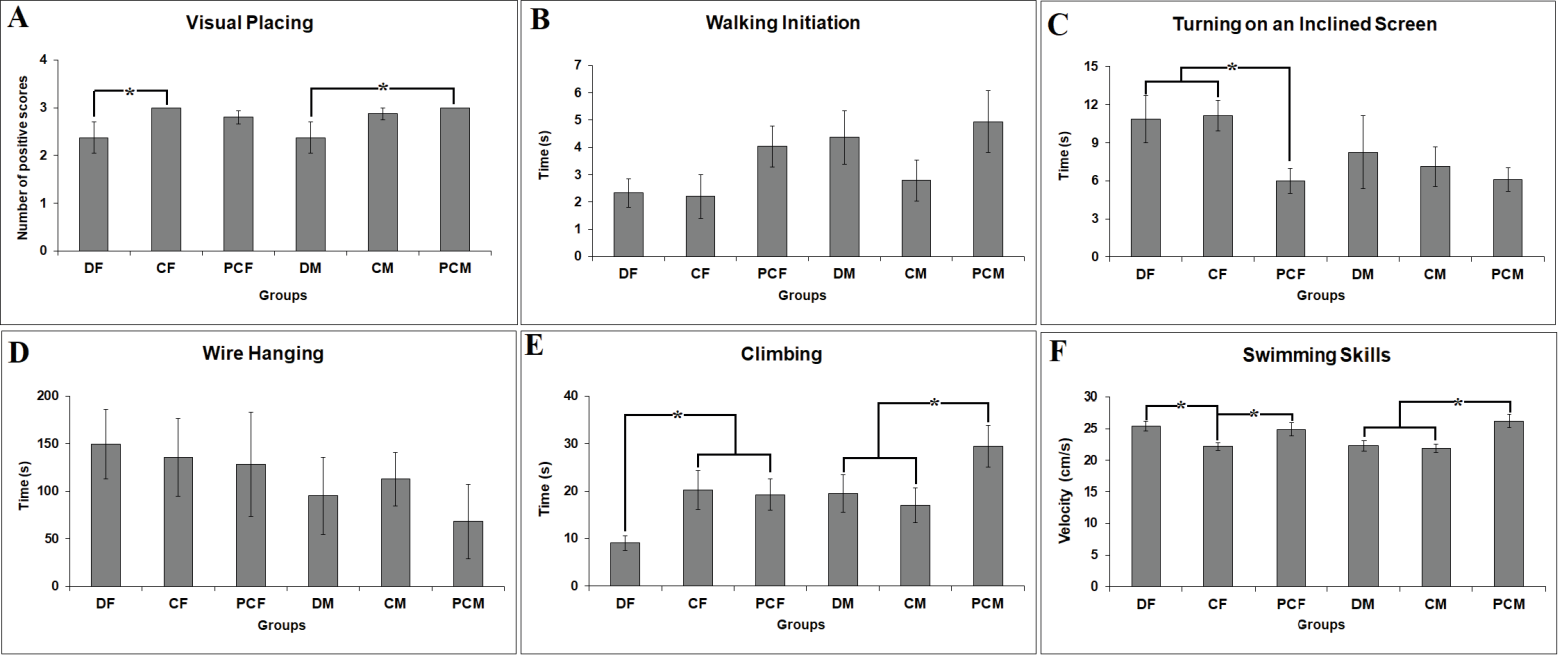
Comparison of sensory motor parameters such as (A) visual placing, (B) walking initiation, (C) turning on an inclined screen, (D) wire hanging, (E) climbing, and (F) swimming skills in fetal diclofenac-treated female (DF) and male (DM) rat pups in comparison with male (CM and PCM) and female (CF and PCF) sham and pure control rat pups. Error bars denote SEM. Asterisks indicate significant difference at P ≤ 0.05.

In the visual placing task, the pup was slowly moved towards the edge of the table, holding it by its back. Extension of the forelimbs was counted as a positive score for three trials. Between-group differences yielded marginally significant differences (F_(5:51)_ = 2.280, P = 0.062). Most of the pups in both control groups had positive scores after 3 trials, but the number of positive scores decreased in the DS-treated pups, both in females and males. Therefore, the D group pups showed worse performance than both control pups (P = 0.029 and P = 0.079 for female and male groups, respectively) (Figure 1A). In the walking initiation test, the time spent for the pup to walk one body length was recorded and there was an insignificant between-group difference (F _(5:51)_ = 1.755, P = 0.141) (Figure 1B). In addition, between-group differences to turn on an inclined (45°) screen after being placed faced downwards on the center of the screen and to fall down after being placed to hang on a horizontal wire yielded insignificant differences (F _(5:51)_ = 2.015, P = 0.094 and F _(3:31)_ = 0.935, P = 0.432, respectively). In the DS-treated group (P = 0.035) and the sham control group (P = 0.027), the time of turning on an inclined screen was higher than the time in the pure control group (Figure 1C). In the wire hanging, the shortest latency to fall was observed in the males of the D group; however, due to high individual variation, this difference was insignificant (Figure 1D). In the clasping the limbs test, in which the rat was suspended by its tail and a positive score was given if its fore and hindlimbs were extended away from the body, all animals except one got positive scores. There was a significant between-group difference (F _(5:51)_ = 3.261, P = 0.013) in climbing a vertical wire mesh screen after being placed at the screen’s bottom at 1/3 of the screen length. The females of the D group were significantly faster compared with the sham control (P = 0.045) and pure control groups (P = 0.053). Both intubated male groups (D and C males) were also significantly slower than males of the PC group (P ≤ 0.05) (Figure 1E). Finally, the swim velocity was significantly different among groups (F _(5:50)_ = 4.594, P = 0.002). The swim velocity of D group females was significantly higher than that of sham control group females (P = 0.018), and, interestingly, C and D group males were significantly slower than PC males in swimming (P = 0.001, and P = 0.004, respectively) (Figure 1F).

### 3.2. Anxiety tests

**Figure 2:**
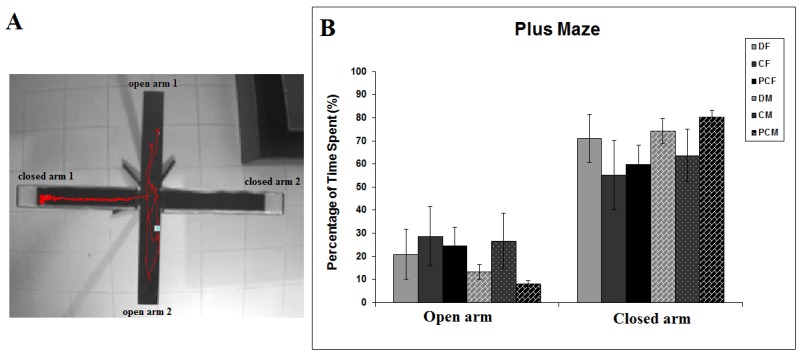
(A) Representative picture of video tracking system showing trajectory of a rat during 5 min. (B) Comparison of the animal’s behavior in the elevated plus maze test as a function of sex (male vs. female) and treatment (D, C, and PC). The bars represent mean percentage of time spent in open and closed arms of the plus maze. Error bars denote SEM.

Representative pictures of the performance of rats are presented in Figure 2A. In the plus maze test, there was a trend among the groups to spend relatively less time in the open arms of the maze (Figure 2B). Nevertheless, the treatment effect was insignificant when one-way ANOVA was performed for each arm independently (F _(5:50)_ = 0.906, P = 0.486 for open arms; F _(5:50)_ = 1.094, P = 0.377 for closed arms).

**Figure 3:**
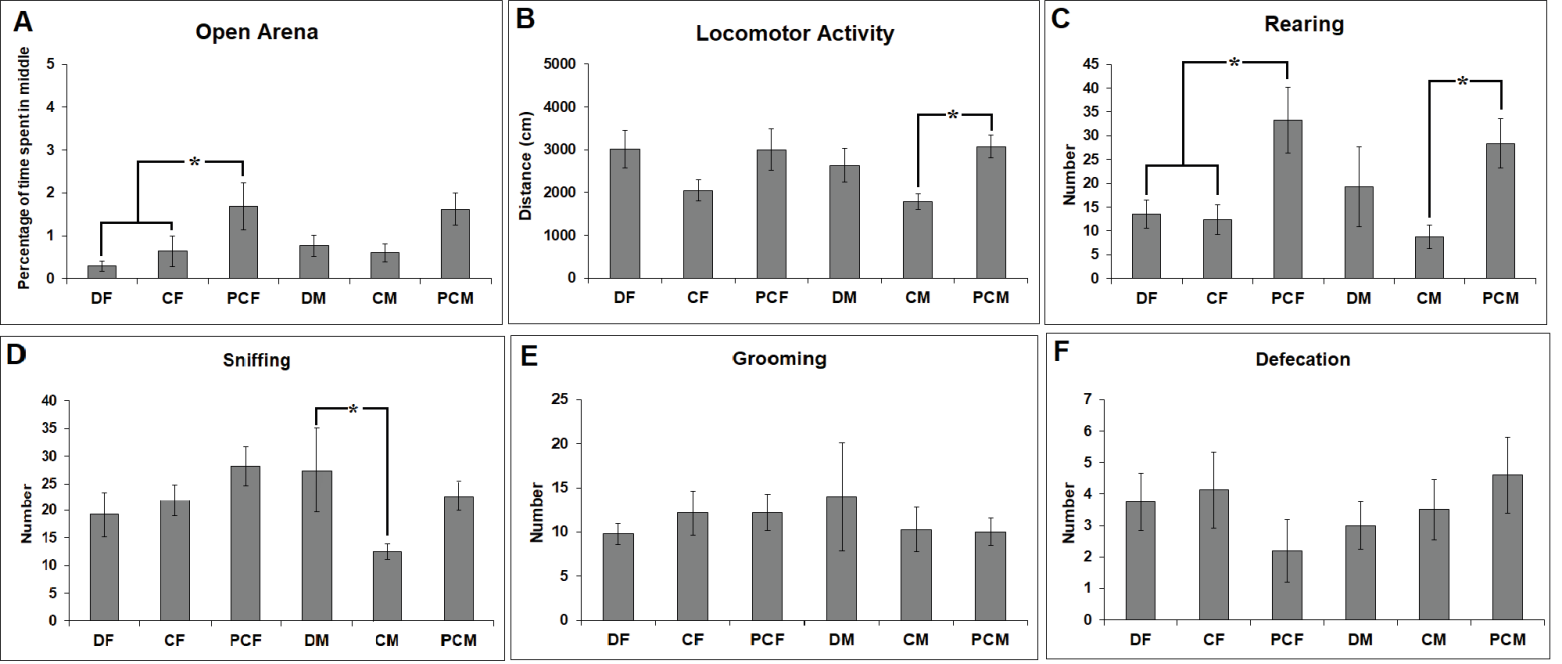
Comparison of the animal’s behavior in the open field test as (A) the mean time spent in the middle zone of the open field, (B) the mean distance moved in the outer and the inner zone of the open field to measure the locomotor activity, and (C) rearing, (D) sniffing, (E) grooming, and (F) defecation scores of animals in the open field during the total 10-min testing period for all groups. Error bars denote SEM. Asterisks indicate significant difference at P ≤ 0.05.

In addition, Figure 3A presents the mean percentage of time spent in the inner zone of the open field, an index of anxiety level. Between-group differences yielded significant results according to one-way ANOVA with group as an independent factor (F _(5:51)_ = 2.589, P = 0.038). The pups in the D group spent significantly more time in the outer zone compared to the pups in the sham control group. The anxiety level of females in the D group was particularly higher than the anxiety level of females in the PC group (P = 0.010). In addition, the total distance moved in the open field was used as an index of locomotor activity and exploratory drive, and there was a significant between-group difference in this parameter (F _(5:48)_ = 2.368, P = 0.05). However, a significant difference was only observed between PC males and C males (Figure 3B). Parallel to the level of anxiety, the rearing activity was different among the groups (F _(5:51)_ = 3.283, P = 0.013). Post hoc tests revealed lower numbers of rearings in the female treatment groups compared to the pure female control group (P = 0.013 for D and P = 0.011 for C females) (Figure 3C). In the male D group, there was an increase in sniffing behavior compared to control males (P = 0.017) (Figure 3D). There were no significant between-group differences in the grooming and defecation scores, indices of anxiety. (Figure 3E-3F).

### 3.3. Learning and memory tests

**Figure 4:**
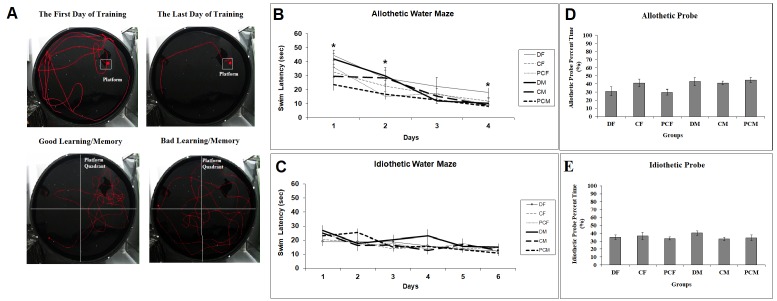
(A) Representative picture of video tracking system showing trajectory of a rat during the training and probe trial. Mean swim latency calculated for the first 4 days of MWM training with allothetic cues (B) and six consecutive days of MWM training without distal visuospatial cues in the idiothetic paradigm (C). Mean percentage of time spent in the platform quadrant on the probe trial under allothetic cues (D) and idiothetic cues (E). Error bars denote SEM. Asterisks indicate significant difference at P ≤ 0.05.

Representative pictures of the performance of rats are presented in Figure 4A. In the MWM training sessions, there was a decrease in the time spent in the maze to reach the hidden platform in all groups (Figure 4). In the allothetic paradigm, there was a significant difference in task acquisition between groups (F _(5:45)_ = 5.070, P = 0.001), while there was no difference in the task acquisition of the idiothetic paradigm F _(5:45)_ = 0.831, P = 0.535). Two-way repeated measure ANOVA (treatment × day) showed that there was a significant day effect for both paradigms F _(3:135)_ = 57.712, P ≤ 0.001 in the allothetic paradigm; F _(5:225)_ = 9.157, P ≤ 0.001 in the idiothetic paradigm). In addition, day × group interactions for both paradigms were insignificant. On each training day, one-way ANOVA showed that, compared to the controls, D group rats had significantly worse performance on the first, second, and fourth training days under allothetic cues F _(5:50)_ = 4.069, P = 0.004; F _(5:50)_ = 2.675, P = 0.034; F_(5:50)_ = 3.842, P = 0.006, respectively), but no differences were observed between groups on each day for the idiothetic paradigm. Females of the D group showed a slower rate of learning compared to both controls for all days in the allothetic paradigm (P ≤ 0.05). Male pups of the D group also showed worse performance compared to both controls on the first two days in the allothetic paradigm (P ≤ 0.05) (Figure 4B). Under idiothetic training conditions with distal visuospatial cues absent, males of the D group showed a slower rate of learning compared to both controls only on the fourth day (P = 0.021) (Figure 4C). In the probe trial of the allothetic paradigm (F _(5:50)_ = 2.587, P = 0.039), females of the D group showed marginally lower (P = 0.059) place preference for the platform quadrant compared to males of the D group (Figure 4D). However, there was no difference between the groups in the place preference retention test (F _(5:50)_ = 0.714, P = 0.616) (Figure 4E).

**Figure 5:**
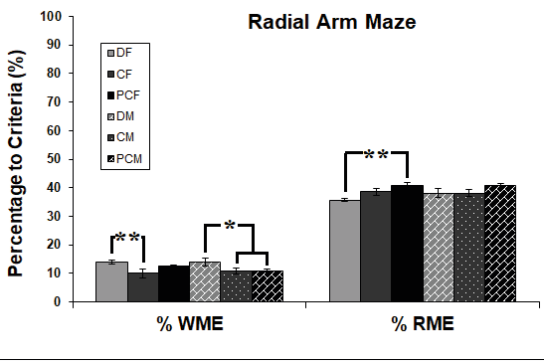
Comparison of the percentage of working and reference memory errors among groups at each of three training stages. Error bars denote ±SEM. Asterisks indicate significant difference at P ≤ 0.05.

In addition, the frequency of errors in reference memory (RME) and working memory (WME) were calculated for each group independently for the arbitrary performance criterion of three consecutive daily trials with 5 out of the first 6 choices being baited arms. One-way ANOVA showed that there were significant differences between groups in the percentage of errors (both WME and RME) for the criterion (F _(5:49)_ = 2.840, P = 0.026 and F _(5:49)_ = 2.940, P = 0.022, respectively). According to the post hoc test, there was a higher percentage of WME in both the females (P = 0.009 for C females) and males (P = 0.038 and P = 0.027 for C and PC males, respectively) of the D group compared to both control groups (Figure 5). Interestingly, the percentage of RME of D group females was lower than that of PC females (P = 0.002) with an insignificant difference between the male groups (Figure 5).

## 4. Discussion

The dosage, timing, and route of drug exposure are the main factors for the determination of the severity of fetal exposure to drugs for the development of the central nervous system. During prenatal development, these drugs modulate the proteins that are components of the neurotransmission of brain development, and these effects may sometimes appear during adult life as behavioral defects. Many substances such as alcohol and nicotine can alter the developmental pathways and produce severe deficiencies in the brain (Thompson et al., 2009; Elibol-Can et al., 2014). On the other hand, during gestation, mothers have to use some prescribed medications to treat neurological alterations, psychiatric problems, or maternal pregnancy disorders. However, there are a limited number of studies examining the long-term functional implications of these drug treatments. Therefore, the current study’s aim was to examine whether prenatal administration of DS, a nonsteroidal antiinflammatory drug, affects behavioral performance in adult life. 

In previous studies, it was observed that fetal exposure to DS to Wistar rats during the equivalent of the 2nd and 3rd trimesters resulted in a significant decrease in the count of cerebellar Purkinje cells, which are mostly involved in motor function, and dentate granular neurons, but not CA pyramidal cells, which have a role in learning and memory (Gokcimen et al., 2007; Ragbetli et al., 2007; Yurt et al., 2018). In the current study, these morphological changes correlated with some decreased sensory motor functions observed in both males and females, and some decline in cognitive functions manifested by slower rates of place learning and decline in working memory in both sexes. 

In the current study, a decrease was observed in the visual placing of pups in the D group, suggesting a DS-related defect in the neocortex (Braun, 1966). Delays in the onset of the visual placing reflex as a teratogenic effect were also observed with other molecules such as ethanol (Ciociola et al., 1988). On the contrary, other antiinflammatory drugs would improve the visual deficiencies by reducing the delays in visual placing, especially in pathological situations (Borzeix and Cahn, 1987). In parallel, the climbing time was longer in the D group pups compared to before controls, pointing to a DS-dependent developmental delay related to sensory motor functions. These DS-related sensory motor deficiencies might suggest that DS exposure during development produces a detrimental effect on motor functions in adult life. Interestingly, pups born from sham control dams manifested a trend towards a decrease in motor functions in some tasks. As stated previously, there are experimental data on the effects of prenatal stress on brain functions (Nazeri et al., 2015). However, in the present experiments, an effect of DS was observed on some sensory motor performances, suggesting that DS has a negative effect on the development of motor functions.

In the present study, no effect of fetal DS exposure per se on emotionality (anxiety) was noted in the plus maze test. On the other hand, an injection-related increase in anxiety was observed in the open field due to a decrease in time spent in the middle area and the number of rearings in the female D and C group pups. In a previous study, investigators also observed administration of diclofenac to confirm that its role in stress did not modify serotonin administration in rats (Bhattcharyya and Sur, 1999). On the other hand, Borges et al. observed that diclofenac was effective on pain-related anxiety in monoarthritis-induced rats (Borges et al., 2014). Furthermore, in a retrospective analysis done on population scale, diclofenac showed a distinctive effect on anxiety by 2 times (Makunts et al., 2018). Not observing an effect of DS per se on anxiety in our study may be related to the adverse effect of injection-related prenatal stress on the developing brain, as stated in previous studies (Elibol-Can et al., 2014; Boschen et al., 2015). For male pups, the amount of sniffing, as an exploration behavior, was higher in the D group than the C group. As stated previously, an increase in sniffing behavior may be a sign of stress (Boukouvalas et al., 2008). Therefore, DS administration in the prenatal period increases stress in the pups rather than representing an anxiolytic effect. 

Based on the present data, it is suggested that prenatal exposure to DS may induce some neurodevelopmental aberrations in animals such as a decline in learning capacity. In addition, DS treatment disrupted the working memory of pups of both sexes. On the other hand, a better performance was observed in the females of the D group in terms of reference memory, while there was no effect of DS on long-term spatial memory. In a previous study, 5-day diclofenac treatment administered to adult animals also decreased short-term memory without any effects on long-term memory (Emad et al., 2017). The slight decrease in learning performance and working memory may be related to the detrimental effect of prenatal DS administration on the hippocampal neuron number. In previous studies, it was observed that exposure to DS prenatally caused a decrease in the number of cells in the dentate gyrus of the hippocampus (Yurt et al., 2018). The memory-enhancing capacity of diclofenac, being a COX inhibitor, was also postulated in previous experiments performed with animals whose memories were suppressed by an amyloid-beta infusion (Kotilinek et al., 2008). This improvement may occur because of diclofenac’s effects on acetylcholinesterase and Na+, K+, and ATPase activities, which are important factors for memory (Muraoka and Miura, 2009; Ali and Arafa, 2011). It was also suggested that COX inhibition also reduces the oxidative stress and enhances the memory (Mahmood et al., 2009; Emad et al., 2017). In addition, DS decreases arachidonic acid and prostaglandins, which have a detrimental role in memory impairment by dual activity on the cyclooxygenase pathway and lipoxygenase pathway (Ku et al., 1986; Matsumoto et al., 2004). However, we observed the positive effect of DS only on female pups due to the difference between males and females in response to the formation of different ion gradients due to different expressional levels of proteins (Fraser and Sarnacki, 1989; Wang et al., 2000; Ali and Arafa, 2011). 

In conclusion, in the present study, it was observed that DS administration in the prenatal period produced a developmental delay related to sensory functions with an increase in stress. In addition, prenatal exposure to DS may induce some neurodevelopmental aberrations such as a decline in learning capacity and working memory. On the other hand, better performance was observed in the female D group pups in terms of reference memory, suggesting that the female rats were more sensitive than males to the prenatal effects of DS. In agreement with the present data, prenatal DS exposure during the equivalent of the second trimester may increase the risk of later psychopathologies such as developmental coordination disorders.

## Acknowledgment

The authors thank Prof Dr Süleyman Kaplan for his help and suggestions at the beginning of this study.
